# Pdx-1 or Pdx-1-VP16 protein transduction induces β-cell gene expression in liver-stem WB cells

**DOI:** 10.1186/1756-0500-2-3

**Published:** 2009-01-09

**Authors:** Juliette Cuvelier Delisle, Lionel Martignat, Laurence Dubreil, Pierre Saï, Jean-Marie Bach, Vanessa Louzier, Steffi Bösch

**Affiliations:** 1 UMR 707 IECM ENVN, F-44307 Nantes, France; 2 UMR 707 IECM INRA, F-44307 Nantes, France; 3 UMR 707 IECM Nantes University, F-44307 Nantes, France; 4 UMR 703 ENVN, F-44307 Nantes, France; 5 UMR 703 INRA, F-44307 Nantes, France

## Abstract

**Background:**

Pancreatic duodenal homeobox-1 (*Pdx-1*) or *Pdx-1-VP16 *gene transfer has been shown to induce *in vitro *rat liver-stem WB cell conversion into pancreatic endocrine precursor cells. High glucose conditions were necessary for further differentiation into functional insulin-producing cells. Pdx-1 has the ability to permeate different cell types due to an inherent protein transduction domain (PTD). In this study, we evaluated liver-to-pancreas conversion of WB cells following Pdx-1 or Pdx-1-VP16 protein transduction.

**Findings:**

WB cells were grown in high glucose medium containing Pdx-1 or Pdx-1-VP16 recombinant proteins for two weeks. β-like cell commitment was analysed by RT-PCR of pancreatic endocrine genes. We found that WB cells in high glucose culture spontaneously express pancreatic endocrine genes (*Pdx-1, Ngn3, Nkx2.2, Kir6.2*). Their further differentiation into β-like cells expressing genes related to endocrine pancreas development (*Ngn3, NeuroD, Pax4, Nkx2.2, Nkx6.1, Pdx-1*) and β-cell function (*Glut-2, Kir6.2, insulin*) was achieved only in the presence of Pdx-1(-VP16) protein.

**Conclusion:**

These results demonstrate that Pdx-1(-VP16) protein transduction is instrumental for *in vitro *liver-to-pancreas conversion and is an alternative to gene therapy for β-cell engineering for diabetes cell therapy.

## Background

The difficulties encountered in obtaining sufficient supply of transplantable β-cells is a major problem in cell therapy of type I diabetes. Liver may be a potential source of cells for β-cell engineering. Indeed, liver and pancreas derive from the same endodermal region during embryogenesis [1] and hepatocytes and β-cells share similar built-in glucose-sensing systems.

Among transcription factors involved in pancreatic β-cell specification, Pdx-1 plays a central role. All progenitors of the endocrine as well as the exocrine pancreas express Pdx-1 [2,3]. In the adult, Pdx-1 expression is mainly restrained to β-cells where it regulates important β-cell functions like *insulin *transcription. Several *in vitro *studies, using viral or stable plasmid gene transfer, show that *Pdx-1 *expression in hepatic cells results in reprogrammation into insulin producing cells [4-7]. Fusion of Pdx-1 to the VP16 activation domain from *Herpes simplex *virus (Pdx-1-VP16) leads to more efficient liver-to-pancreas conversion than Pdx-1 alone [8-12]. Stable mouse *Pdx-1 *or *Pdx-1-VP16 *gene transfection initiates conversion of rat epithelial liver stem-like WB cell line into pancreatic endocrine precursor cells [13,14]. In these cells, long-term high glucose (HG) culture *in vitro *is necessary for further pancreatic endocrine differentiation.

Safety of gene therapy remaining a prime concern, protein transduction offers a more secure alternative to induce stem cell differentiation. Indeed, protein transduction domains (PTD) allow proteins to translocate across the cytoplasmic membrane. Due to an Antennapedia-like PTD in its structure, Pdx-1 protein can permeate different cell types and induces insulin expression in pancreatic ducts [15,16]. However, in human embryonic stem cells, the adjunction of a PTD domain derived from the HIV TAT protein (TAT) is necessary for Pdx-1 cell transduction [17]. Up to now, no study using protein transduction has achieved liver-to-endocrine pancreas conversion *in vitro*.

Here, we evaluate if Pdx-1(-VP16) proteins have the capacity to induce a pancreatic endocrine shift in WB cells. To achieve this, we treat WB cells with Pdx-1 or Pdx-1-VP16 proteins, containing their own PTD, or fused to the PTD of TAT.

## Methods

### Recombinant proteins synthesis

Full-length mouse *Pdx-1 *and *Pdx-1-VP16 *open reading frames were cloned into pET28b-TAT-v2-1 expression plasmid containing the HIV TAT protein PTD (kind gift from S. Dowdy), in order to construct Pdx-1, Pdx-1-VP16, TAT-Pdx-1 and TAT-Pdx-1-VP16. PTD_Pdx-1_-eGFP was constructed by fusing the PTD of Pdx1 (RHIKIWFQNRRMKWKK) to eGFP and subsequent cloning into pET28b-TAT-v2-1. TAT-eGFP was constructed by insertion of eGFP into pET28b-TAT-v2-1. eGFP was cloned into pET21a(+) expression vector (Novagen, WI, USA). See Additional data 1 for more details.

Recombinant proteins were produced according to Studier's method of auto-induction [18]. Proteins were purified by Ni^2+ ^affinity chromatography on Protino Ni-TED resin (Macherey-Nagel, France), diafiltrated on Centricon-Plus-20 centrifugal filter devices (Millipore, France), and stored at -20°C in PBS/pH8.0/25%glycerol.

### Luciferase assay

The RIP2-reporter gene was constructed by cloning the [-683 bp, +11 bp] 5' flanking region of rat insulin-II gene into the EcoRV-site of pGL4.10 [*luc2*] (firefly luciferase) (Promega, France).

18 × 10^3 ^HepG2 cells/well were seeded onto a 96-well plate and grown 24 h in 10% FCS DMEM (5% CO_2_, 37°C). 0,12 μg of RIP2-reporter and 0,13 μg of HSV-TK-hRluc control vector (renilla luciferase, pGL4-74, Promega) were co-transfected in these cells using JetPEI reagent (Polyplus-Transfection, France). 12 h after transfection, the medium was replaced with medium containing 5 μM protein or storage buffer for negative controls. 36 h later, cells were assayed for luciferase activities using Dual-Glo-Assay-System (Promega) and a scintillation counter (MicroBeta-Trilux, Wallac/Perkin-Elmer, France).

### WB cell culture and protein treatment

WB-F344 cells, kindly provided by N. Malouf [19], were grown in low glucose (LG) medium: 10% FCS RPMI-1640 (11 mM glucose, Invitrogen, France) (5% CO_2_, 37°C).

To evaluate transduction efficiency, 50 × 10^3 ^WB cells/well were seeded onto a 24-well plate. 24 h later, the medium was replaced with fresh medium containing 15 μM eGFP, PTD_Pdx-1_-eGFP, or TAT-eGFP proteins and incubated for another 24 h before confocal microscopy analysis.

To evaluate effects of Pdx-1(-VP16) proteins, 25 × 10^3 ^WB cells/well (passage 15) were seeded onto a 96-well plate with medium adjusted to HG concentration (25 mM D-glucose, Sigma-Aldrich). 12 h later, the medium was replaced by HG medium containing 1 μM protein (Pdx-1 (n = 4), TAT-Pdx-1 (n = 4), Pdx-1-VP16 (n = 5), or TAT-Pdx-1-VP16 (n = 5)) or storage buffer for HG control cells (n = 5). The medium was replaced every 3–4 days. Control cells in LG medium (n = 5) were also grown. To favour differentiation rather than proliferation, WB cells were treated for 2 weeks without being trypsined. Then mRNAs were collected and analysed by RT-PCR. Each sample (n) represents one culture well.

### Laser scanning confocal microscopy

Cells were treated 10 minutes with trypsin/EDTA, washed with PBS, stained with Vybrant-CM-Dil (1:200 dilution in PBS, Molecular Probes), and washed twice. eGFP signal (λ_exc _488 nm, λ_em _507 nm) and CM-Dil staining (λ_exc _553 nm, λ_em _570 nm) were examined by laser scanning confocal microscopy (Nikon TE-2000, France).

### RT-PCR analysis

mRNAs were isolated with the Dynabeads-mRNA-Direct Kit (Invitrogen). First-strand cDNA was synthesized using M-MuLV reverse transcriptase (Promega) and random pentadecamer primers. The resulting cDNA was amplified for 35 cycles [94°C 30 sec, 59/60°C 30 sec, 72°C 40 sec] with RedTaq DNA Polymerase (Sigma-Aldrich) on a 9700 thermocycler (Applied-Biosystems, France) using primers listed in Additional data 2.

## Results

### Pdx-1 and TAT PTDs transduced HepG2 and WB cells

**Figure 1:**
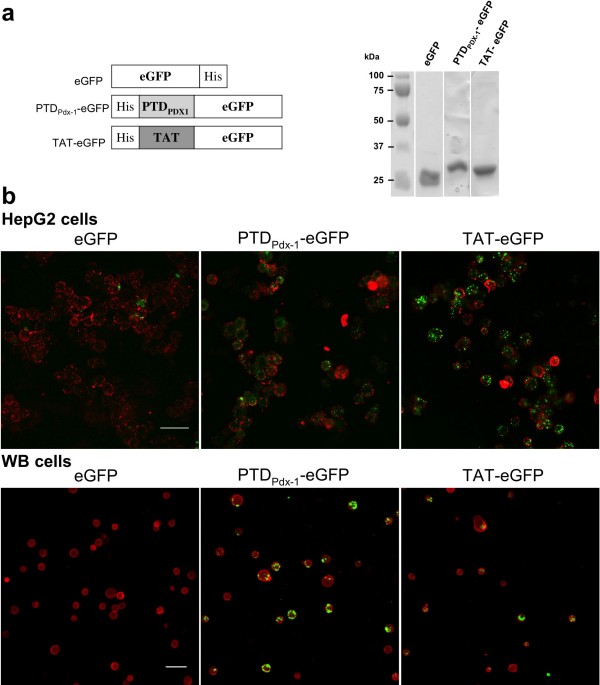
**PTD_Pdx-1 _and TAT fusion protein synthesis and their ability to transduce HepG2 and WB cells**. (a) Schematic structure (top panel) and purity (bottom panel) of fusion proteins. PTD_Pdx-1 _and TAT represent protein transduction domains of the Pdx-1 protein and the HIV TAT protein, respectively. His represents hexahistidine tag used to purify proteins by His-tag affinity chromatography. Purified proteins were run on a SDS-PAGE gel (8%) stained with Coomassie blue. Molecular weights are 28 kDa for eGFP, 31 kDa for PTD_Pdx-1_-eGFP and TAT-eGFP. (b) Observation by confocal microscopy of eGFP fluorescence in HepG2 and WB cells treated for 24 hours with 15 μM PTD_Pdx-1_-eGFP, TAT-eGFP, or eGFP protein lacking PTD. Treated cells were observed by confocal microscopy without being fixed in order to exclude artifactual protein uptake [24]. CM-Dil was used to visualize cytoplasmic membrane (red staining). Scale bars = 50 μm.

To assess PTD_Pdx-1_- or TAT-mediated protein transduction in liver cells *in vitro*, we evaluated the ability of Pdx-1 and TAT PTDs to deliver fusion proteins into WB and HepG2 cells. To achieve this, we produced three recombinant eGFP fusion proteins: PTD_Pdx-1_-eGFP, TAT-eGFP and eGFP alone as a negative control (Fig. 1a). Intracellular eGFP fluorescence was detected in cells treated with PTD_Pdx-1_-eGFP or TAT-eGFP – as punctuated or diffused signals (Fig. 1b). No cellular uptake was detected in cells treated with eGFP protein lacking PTD (Fig. 1b). These results indicated clearly (i) that the efficiency of HepG2 and WB cell transduction was dependant on the presence of a PTD domain, and (ii) that PTD_Pdx-1_efficiently delivered fusion proteins into cells.

### Pdx-1(-VP16) protein transduction increased insulin promoter activity

**Figure 2:**
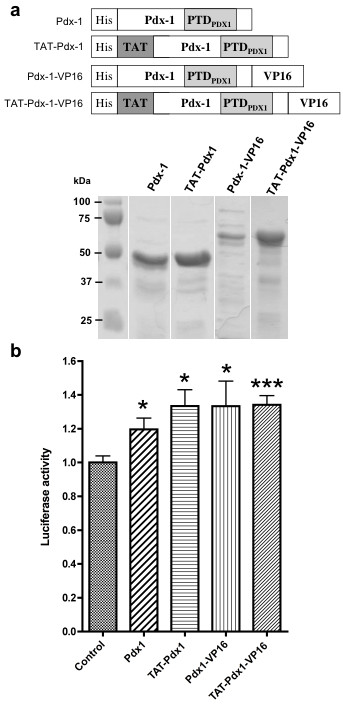
**Synthesis and biological activity of Pdx-1, TAT-Pdx-1, Pdx-1-VP16 and TAT-Pdx-1-VP16 fusion proteins**. (a) Schematic structure (top panel) and purity (bottom panel) of Pdx-1, TAT-Pdx-1, Pdx-1-VP16 and TAT-Pdx-1-VP16 fusion proteins. PTD_Pdx-1 _represents protein transduction domain of the Pdx-1 protein, which is naturally present in the four Pdx-1(-VP16) fusion proteins. TAT represents protein transduction domain of the VIH TAT protein. VP16 is the activation domain of Herpes simplex virus I. His represents hexahistidine tag used to purify proteins by His-tag affinity chromatography. Purified proteins were run on a SDS-PAGE gel (8%) stained with Coomassie blue. Molecular weights are 45 kDa for Pdx-1 and TAT-Pdx-1, 60 kDa for Pdx-1-VP16 and TAT-Pdx-1-VP16. (b) *Insulin *promoter activity after Pdx-1(-VP16) protein transduction. Twelve hours after transient transfection of *insulin *promoter luciferase plasmid, HepG2 cells were treated with 5 μM Pdx-1(-VP16) fusion proteins for 36 hours. Then luciferase activity was measured. Data are expressed as mean + SEM (Control n = 14, Pdx-1 n = 13, TAT-Pdx-1 n = 7, Pdx-1-VP16 n = 12, TAT-Pdx-1-VP16 n = 19). *Insulin *promoter activity of controls was arbitrarily set at 1. Statistical comparisons were performed using a non parametric Mann-Withney test, *: p < 0.05, ***: p < 0.001.

In order to convert WB cells into insulin producing, cells we produced and purified four Pdx-1 proteins: Pdx-1, Pdx-1-VP16, TAT-Pdx-1 and TAT-Pdx-1-VP16 (Fig. 2a). Rat insulin-II promoter activity was significantly increased by treatment with any of the Pdx-1(-VP16) proteins (Fig. 2b). These results showed that these four Pdx-1(-VP16) proteins transduced hepatic cells and, in part, went to the nucleus and activated a β-cell specific promoter. Fusion of TAT to these proteins, which consequently contain two PTDs in their structure, increased insulin promoter activity in an equal manner as PTD_Pdx-1 _alone. Pdx-1 fusion to VP16 activation domain did not further enhance insulin promoter activity.

### Pdx-1, Pdx-1-VP16, TAT-Pdx-1 and TAT-Pdx-1-VP16 proteins induced the expression of insulin and pancreatic-related genes in WB cells

**Figure 3:**
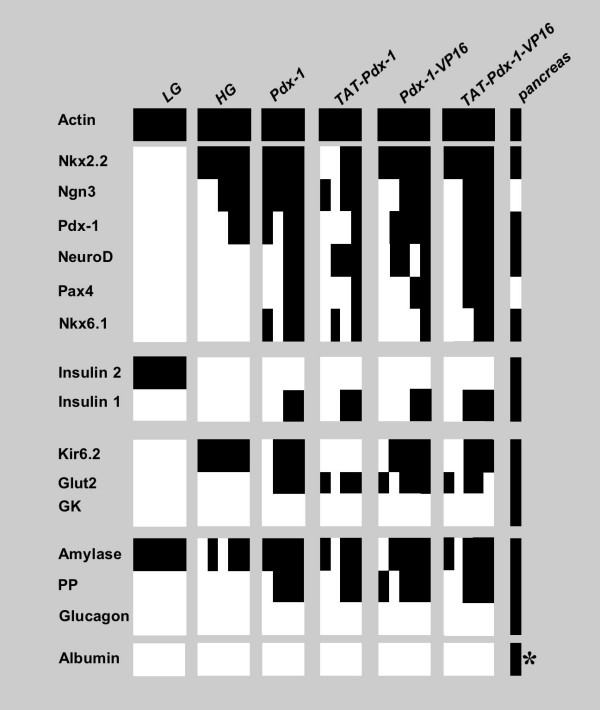
**Gene expression profile of WB-cells treated with Pdx-1(-VP16) or TAT-Pdx-1(-VP16) proteins in high glucose culture**. RT-PCR analysis was performed after two weeks of protein treatment. cDNA from rat pancreas served as control for pancreatic gene expression, and cDNA from rat liver served as positive control for *albumin *expression (*). Each sample is represented by a single column and corresponds to an independent biological repeat of the experiment (LG (n = 5), HG (n = 5), Pdx-1 (n = 4), TAT-Pdx-1 (n = 4), Pdx-1-VP16 (n = 5), TAT-Pdx-1-VP16 (n = 5)). Each gene is represented by a single row of coloured boxes. Black colouring represents RT-PCR positive samples, whereas white colouring represents negative samples. LG: Low Glucose control WB cells; HG: High Glucose control WB cells. (See additional data 3 for original electrophoresis gel images).

Whereas LG control cells expressed *amylase *and weakly *insulin 2*, but none of the other pancreatic genes tested here, HG control cells expressed transcription factors implicated in endocrine pancreatic differentiation: *Ngn3*, *Nkx2.2 *and *Pdx-1 *(Fig. 3). Expression of *Kir6.2 *was also detected. See additional data 3 for original electrophoresis gel images.

Taking advantage of the commitment of WB cells towards an endocrine phenotype in HG culture, Pdx-1(-VP16) protein treatment was performed on WB cells grown in HG medium. Proteins induced the expression of a wide panel of β-cell genes after two weeks of culture: *NeuroD*, *Pax4*, *Nkx6.1*, *insulin 1, Glut-2*. Expression of *Pancreatic Polypeptide (PP)*, a non-β endocrine hormone, was also detected. Among different samples, some expressed all of these β-cell genes (*Ngn3, NeuroD*, *Pax4*, *Nkx2.2, Nkx6.1*, *Pdx-1, insulin 1, Glut-2*, *Kir6.2) *and others only some of them. Although *insulin 1 *was expressed, *insulin 2 *expression was not induced by Pdx-1(-VP16) protein treatment. *Glucokinase *(*GK) *was not detected despite using primers amplifying pancreatic as well as hepatic *GK *isoforms.

No pancreatic endocrine gene expression was observed at earlier time points (day 6 and 9 of treatment, data not shown).

The hepatic marker *Albumin *was absent in all conditions tested, including LG control cells.

## Discussion

In the present study, we demonstrate the aptitude of Pdx-1 or Pdx-1-VP16 protein transduction to reprogram hepatic stem-like cells into β-like cells *in vitro*.

First, we verified that the PTD of Pdx-1 and TAT allow transduction into hepatic WB and HepG2 cell lines. Intracellular localization of fusion proteins revealed by confocal microscopy analysis indicate a true uptake of proteins and not mere adherence to the cell surface. Furthermore, transduced recombinant Pdx-1, TAT-Pdx-1, Pdx-1-VP16 and TAT-Pdx-1-VP16 proteins exert biological activity on an insulin promoter reporter system. Pdx-1 fusion to VP16 activation domain does not further enhance insulin promoter activity consistent with previous findings [10]. Our results confirm reports of Pdx-1 transduction [15,20] and are the first demonstration of Pdx-1-VP16 protein transduction.

Pancreatic differentiation experiments were conducted in an HG environment. In fact, long-term HG culture furthers liver cell commitment towards a pancreatic fate [4,6,13,14,21]. Previous studies of *Pdx-1(-VP16) *expression in WB cells do not distinguish between respective contributions of transgene expression and HG culture on differentiation. Here, we show that HG culture alone converts WB cells into pancreatic endocrine precursor cells. In contrast to LG cultures, HG cultures express four pancreatic endocrine genes: *Ngn3, Nkx2.2, Pdx-1 *and *Kir6.2*. These results concur with Yang *et al. *study, where confluent culture of hepatic oval stem cells for 2 months in HG medium induces conversion into insulin-producing cells [21]. *Kir6.2 *expression is at odds with previous reports where *Kir6.2 *was detected in WB cells overexpressing *Pdx-1 *or *Pdx-1-VP16 *genes only (i) after 2–3 months in HG culture [14], (ii) 40 days post-transplantation into diabetic mouse [13], or (iii) after *Pax4 *co-expression [22].

Pdx-1, TAT-Pdx-1, Pdx-1-VP16, or TAT-Pdx-1-VP16 proteins further induce expression of *NeuroD, Pax4, Nkx6.1*, *insulin 1 *and *Glut-2 *after two weeks of treatment. Some samples express just a part of these markers or display RT-PCR expression patterns similar to HG controls. Quantitative analysis would help to further nuance pancreatic gene expression in-between these samples, in particular up-regulation of the endogenous Pdx-1 gene. The heterogeneity between samples may be a consequence of: (i) infrequent liver to endocrine pancreas conversion, leading to few pancreatic gene positive cells which may be difficult to detect, (ii) different kinetics of gene expression between wells. According to Tang *et al. *study, our experiments do not reveal more efficient differentiation following Pdx-1-VP16 protein treatment compared to Pdx-1 protein treatment [14]. TAT-mediated transduction does not lead to more advanced differentiation suggesting that containing two PTDs (PTD_Pdx-1 _and TAT) does not increase transduction efficiency. Surprisingly, *insulin 1 *and *Pax4*, two of the pancreatic genes expressed after our protein treatments, are not detected in a previous *in vitro *study on WB cells transduced with *Pdx-1 *or *Pdx-1-VP16 *genes, even after 3 months of HG culture [14]. Here, despite *insulin 1 *expression, neither *insulin 2 *nor *glucokinase *are detected pointing at the possible need for long-term culture in HG medium to obtain mature β-like cells [14]. Moreover, *Ngn3 *expression in wells scoring positive for all other pancreatic genes, including *insulin 1*, suggests the presence of remaining subpopulations of immature pancreatic precursor cells.

Overexpression of *Pdx-1 *or *Pdx-1-VP16 *genes in hepatic cells leads to exocrine as well as a range of endocrine cell types [4,7,8,13,14]. In our study, Pdx-1(-VP16) or TAT-Pdx-1(-VP16) protein treatments result in expression of *PP*, but not of *glucagon *suggesting that Pdx-1(-VP16) transduction in hepatic stem-like cells may lead to endocrine β-cell and non-β-cell phenocopies. *Amylase*, expressed in rat liver or exocrine pancreatic cells, is detected in all conditions tested here [23].

Recently, Koya *et al. *demonstrated that Pdx-1 protein delivery into diabetic mice restores euglycemia mainly through pancreatic β-cell regeneration. The authors observed β-cell gene expression and insulin synthesis in the pancreas and in the liver of treated mice suggesting hepatic insulin contribution to euglycemia [20]. In complement, our findings provide the first direct evidence that Pdx-1(-VP16) protein transduction in conjunction with HG culture reprograms hepatic stem-like cells into cells displaying similarities with β-cells *in vitro*. At a point where strategies for targeted β-cell differentiation begin to surface, our study illustrates how simple exposition to Pdx-1(-VP16) protein in the surrounding medium triggers short-term pancreatic endocrine conversion. This study may contribute to the development of protein transduction therapy, a new concept to induce β-cell differentiation.

## Competing interests

The authors declare that they have no competing interests.

## Authors' contributions

JC, SB and VL contributed to protein synthesis. LD and SB carried out confocal microscopy imaging. JC and VL performed luciferase assay, cell culture, RT-PCR analysis and drafted the manuscript. All authors contributed to the design of the study and interpretation of data. All authors read and approved the final manuscript.

## Supplementary Material

Additional file 1 **Supplementary materials and methods.** Click here for file

Additional file 2 **List of primer information for RT-PCR.** Click here for file

Additional file 3 **Electrophoresis gel of RT-PCR analysis of WB cells treated with Pdx-1(VP16) proteins.** Click here for file
