# Supplementary material for: Pdx-1 or Pdx-1-VP16 protein transduction induces β-cell gene expression in liver-stem WB cells
Source: BMC Res Notes. 2009 Jan 9;2:3. doi: 10.1186/1756-0500-2-3 (PMC2637887; doi:10.1186/1756-0500-2-3)
Supplement: Additional file 1 — Supplementary materials and methods. [file 1756-0500-2-3-S1.pdf]

Supplementary materials and methods

Construction of vectors and purification of recombinant proteins

Full-length mouse *Pdx-1* and *Pdx-1-VP16* open reading frames were amplified by PCR, respectively, from pGEM-T-Pdx-1 and pTTR-pdx1-VP16-Elastase-GFP which contains the VP16 transactivation domain of Herpes simplex virus I fused to the C-terminus of mouse *Pdx-1* (a generous gift from M. Horb). PCR was carried out using : forward Pdx-1 primer with *NdeI* site 5'-GCCACG*CATATG*AACAGTGAGGAG-3' or *BamHI* site 5'-GAGCAC*GGATCC*TAACAGTGAGGAG-3' ; reverse Pdx-1 primer 5'-GCACG*AAGCTT*TCACCCTCAGAC-3' or Pdx-1-VP16 primer 5'-GACCGC*AAGCTT*CAAACTCATCAA-3' with *HindIII* sites. pET28b-TAT-v2-1 expression plasmid containing the HIV TAT protein PTD (named TAT in this study) was kindly provided by S. Dowdy. PCR products were inserted into *NdeI-HindIII* sites of pET28b-TAT-v2-1 to construct Pdx-1 and Pdx-1-VP16, and into the *BamHI-HindIII* site or the blunted *SalI-HindIII* site to construct TAT-Pdx-1 and TAT-Pdx-1-VP16, respectively.

PTD_{Pdx-1}-eGFP was constructed by fusing the PTD of Pdx1 (RHIKIWFQNRRMKWKK) to eGFP as follows : PTD_{Pdx-1} was amplified by PCR on pTTR-Pdx-1-VP16–Elastase-GFP with forward primer featuring *NdeI* site 5'-CCACG*CATATG*AGACACATCA-3' and reverse primer featuring *BamHI* site 5'-ATAT*GGATCC*TTTTTCCACTTCA-3' ; eGFP was amplified from pEGFP1 (Clontech, France) using forward primer with *BamHI* site 5'-TGAT*GGATCC*AGGAGTGAGCAAG-3' and reverse primer with *HindIII* site 5'-CGTG*AAGCTT*TCACTTGTACAGCTC-3'. PTD_{Pdx-1} and eGFP products were inserted into *NdeI-BamHI* and *BamHI-HindIII* sites of pET28b-TAT-v2-1 to yield PTD_{Pdx-1}-eGFP.

TAT-eGFP was constructed by amplification of eGFP from pEGFP1 using forward primer with *BamHI* site 5'-TGAT*GGATCC*GTGAGCAAG-3' and reverse primer with *HindIII* site 5'-CGTG*AAGCTT*TCACTTGTACAGCTC-3' and insertion into blunted *BamHI-HindIII* sites of pET28b-TAT-v2-1.

eGFP was amplified from pEGFP1 with forward primer containing *NdeI* site 5'-CGAT*CATATG*GTGAGCAAGG-3' and reverse primer with *XhoI* site 5'-CATT*CTCGAG*CTTGTACAGCTC-3' and inserted into *NdeI-XhoI* sites of pET 21a (+) expression vector (Novagen, WI, USA).
