# Supplementary material for: Pdx-1 or Pdx-1-VP16 protein transduction induces β-cell gene expression in liver-stem WB cells
Source: BMC Res Notes. 2009 Jan 9;2:3. doi: 10.1186/1756-0500-2-3 (PMC2637887; doi:10.1186/1756-0500-2-3)
Supplement: Additional file 2 — List of primer information for RT-PCR. [file 1756-0500-2-3-S2.pdf]

List of primer information for RT-PCR

Genes	Forward primer	Reverse primer	PCR size (bp)	GenBank Accession Number	Tm (°C)
**Actin**	AGCCATGTACGTAGCCATCC	CTCTCAGCTGTGGTGGTGAA	228	V01217	60
**Ngn3**	GAGTGGGTGGGCGTACTCTA	TTGGAACTGAGCACTTCGTG	186	NM_021700	59
**Pdx-1**	GGCTTAACCTAAACGCCACA	GGGACCGCTCAAGTTTGTAA	247	NM_022852	59
**NeuroD**	GGATGATCAAAAGCCCAAGA	GCAGGGTACCACCTTTCTCA	163	NM_019218	59
**Nkx2.2**	GGGGTTTTCAGTCAAGGACA	AGTCCGTGCAGGGAGTATTG	246	XM_345446	60
**Nkx6.1**	ACTTGGCAGGACCAGAGAGA	GGGCTTGTTGTAATCGTCGT	209	NM_031737	59
**Pax4**	AGGACGCTACTACCGCACAG	GGTACAAAGCCCTTCAGCAC	169	NM_031799	60
**Insulin 1**	CACCTTTGTGGTCCTCACCT	CCAGTTGGTAGAGGGAGCAG	232	NM_019129	59
**Insulin 2**	CATCAGCAAGCAGGTYATTG<br>Y : C or T	CACTTGTGGGTCCTCCACTT	216	V01243	60
**Glut2**	TAAGGGGCACTGAGGACATC	TGCCAGCTGTCTGAAAAATG	216	J03145	60
**Glucokinase**	CAGTGGAGCGTGAAGACAAA	CTTGGTCCAATTGAGGAGGA	216	NM_012565	60
**Kir6.2**	GTAGGGGACCTCCGAAAAAG	TTGGAGTCGATGACGTGGTA	188	NM_031358	60
**Glucagon**	TCGTGGCTGGATTGTTTGTA	GTGAATGTGCCCTGTGAATG	160	NM_012707	60
**PP**	CTATCCACTTGGGTGGCTCT	ACCTAGGCCTGGTCAGTGTG	157	NM_012626	59
**Amylase**	GGCCTTCTGGATCTTGCAC	TCCTTGGGAGAACCATTTTG	186	J00703	60
**Albumin**	AAAGCCACCTGCTTTCAAGA	TCACAGCACCCGTCATACAT	221	BC091391	60

Whenever possible, primers were designed to cross intron-exon boundaries in order to avoid genomic DNA amplification. For other primers, RT enzyme was omitted in the reverse transcriptase step of samples serving as negative controls (data not shown).
