# Supplementary material for: Pdx-1 or Pdx-1-VP16 protein transduction induces β-cell gene expression in liver-stem WB cells
Source: BMC Res Notes. 2009 Jan 9;2:3. doi: 10.1186/1756-0500-2-3 (PMC2637887; doi:10.1186/1756-0500-2-3)
Supplement: Additional file 3 — Electrophoresis gel of RT-PCR analysis of WB cells treated with Pdx-1(VP16) proteins. [file 1756-0500-2-3-S3.pdf]

Electrophoresis gel of RT-PCR analysis of WB cells treated with Pdx-1(VP16) proteins.

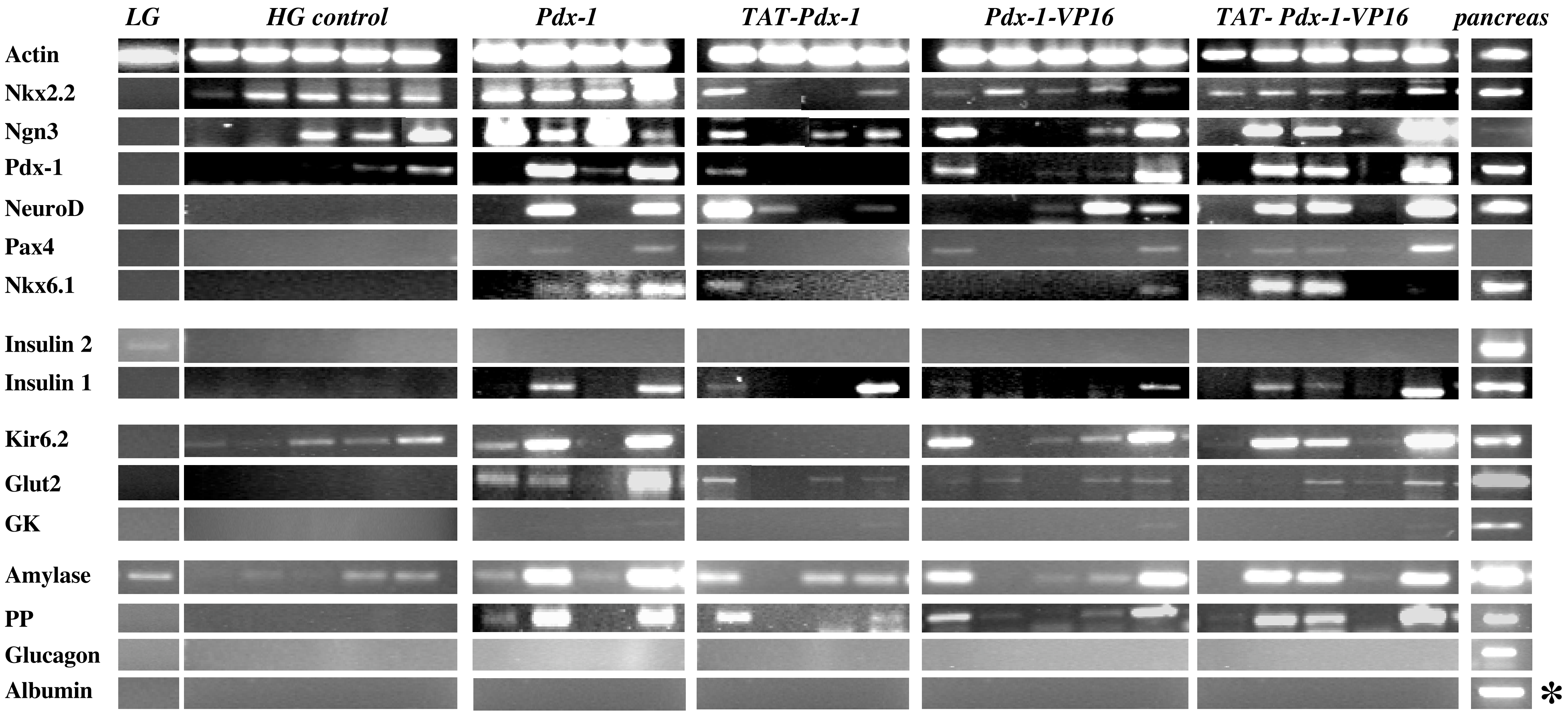

RT-PCR analysis was performed after two weeks of protein treatment. cDNA from rat pancreas served as control for pancreatic gene expression, and cDNA from rat liver served as positive control for *albumin* expression (*). LG : Low Glucose control WB cells ; HG : High Glucose control WB cells. Each column represents independent biological repeat of the experiment.
